# Predictive Value of the Model of End-Stage Liver Disease in Cirrhotic Patients with and without Spontaneous Bacterial Peritonitis

**DOI:** 10.1155/2012/539059

**Published:** 2012-02-22

**Authors:** Bledar Kraja, Marsela Sina, Iris Mone, Fatjona Pupuleku, Adriana Babameto, Skerdi Prifti, Genc Burazeri

**Affiliations:** ^1^University Clinic of Gastrohepatology, University Hospital Center “Mother Theresa”, Dibra Street No. 370, Tirana, Albania; ^2^Department of Laboratory Medicine, University Hospital Center “Mother Theresa”, Tirana, Albania; ^3^Institute of Statistics (INSTAT), Tirana, Albania; ^4^Department of International Health, CAPHRI School for Public Health and Primary Care, Faculty of Health, Medicine and Life Sciences, Maastricht University, Maastricht, The Netherlands

## Abstract

*Objective*. We aimed to assess the predictive value of the model of end-stage liver disease (MELD) in hospitalized cirrhotic patients with and without spontaneous bacterial peritonitis (SBP) and fatal outcome. *Methods*. A cross-sectional study included 256 consecutive patients (199 men and 57 women) diagnosed with cirrhosis and ascites who were hospitalized at the University Hospital Center in Tirana from January 2008 to December 2009. SBP was defined as a neutrophil count of ≥250 cells/mm^3^ in ascitic fluid. MELD score was based on laboratory parameters determined by UNOS Internet site MELD calculator. *Results*. In multivariable-adjusted logistic regression models controlling for age, sex, diabetes, and etiology, there was evidence of a positive association of SBP with MELD score: the odds ratio (OR) for SBP for one unit increment of MELD score was 1.06 (95% Cl = 1.02–1.09). MELD score was significantly higher in fatal cases than nonfatal patients (mean age-adjusted score was 32.7 versus 18.4 overall; 34.8 versus 18.0 in SBP patients, and 32.0 versus 18.5 in non-SBP patients; all *P* < 0.001). *Conclusions*. In this Albanian sample of hospitalized cirrhotic patients, MELD score was confirmed as a significant predictor of both SBP and fatal outcome.

## 1. Introduction

Spontaneous bacterial peritonitis (SBP) is a common and serious infection occurring in patients with cirrhosis and ascites [1] which may be complicated by renal failure, systemic sepsis, and poor survival [2, 3]. SBP originates due to bacteria that translocate across the leaky gut [4, 5]. Uncontrolled bacterial growth in ascites fluid then develops as a result of an impaired host immune response [6, 7]. Numerous studies suggest that 10–30% of hospitalized patients with cirrhosis and ascites have SBP, with in-hospital mortality ranging from 20–40% [8–10].

Outcome prediction of patients with chronic end-stage liver disease (ESLD) is very important. In 2001, the Mayo Clinic group suggested a novel model to predict the outcome of patients with ESLD, which they named MELD [11, 12]. In 2003, MELD was formally adopted by the UNOS (United Network of Organ Sharing) for allocation and quality control. It has since been validated in many other aspects of terminal liver disease, including the prediction of prognosis in viral or alcoholic hepatitis [13], risk of mortality among cirrhotic patients [14], outcome following liver transplantation [15], and, recently, also as a prognostic tool for patients with acute liver failure [16].

Because of the significant morbidity and mortality related to SBP, identifying predisposing factors is of great interest. MELD score is a measure of mortality risk in patients with ESLD [17]. Development of ascites and encephalopathy, two complications of ESLD that are not used in the MELD score calculation, has generally correlated with higher MELD scores [18].

Because of low social economic level, poor sanitary conditions, and lack of hepatitis B vaccination of the population over 12-13 years old, the Albanian population is highly infected with hepatitis B virus [19]. In our country we find also a high rate of alcohol abuse [20]. Due to these two main factors, we have an important number of patients with hepatic cirrhosis, SBP, and liver failure. We used MELD score in our hospitalized patients with liver cirrhosis to predict the occurrence of SBP and fatal outcome.

## 2. Methods

### 2.1. Study Population

Our cross-sectional study included 256 consecutive patients diagnosed with cirrhosis and ascites hospitalized at the University Hospital Center in Tirana from January 2008 to December 2009. We excluded patients with (i) cirrhosis and ascitis who had antibiotic treatment or upper gastrointestinal hemorrhage within two weeks prior to admission; (ii) were on treatment with proton pump inhibitors (PPI); (iii) patients with ascitis unrelated to their cirrhosis such as congestive heart failure, malignancy with metastasis; (iv) patients diagnosed with hepatocarcinoma in the course of hospitalization.

### 2.2. Data Collection

Data collected included patient age, sex, etiology of cirrhosis, history for diabetes mellitus, and ascitic fluid analysis. All patients underwent a diagnostic paracentesis on admission. SBP diagnosis was based on neutrophil count in ascitic fluid ≥250 cells/mm^3^. We did not require positive ascites fluid cultures to diagnose SBP. Patients with ascites fluid neutrophil cell counts of <250 cells/mm^3^ were considered not to have SBP according to the recommended guidelines of the *European Association for the Study of the Liver *(http://www.easl.eu/). Paracentesis was carried out with or without ultrasound guidance using a standard sterile technique. All patients included in our study were treated according to the recommended guidelines of the *European Association for the Study of the Liver*. Laboratory data included total serum bilirubin, serum creatinine, and prothrombin time with international normalized ratio (INR). MELD score was based on laboratory parameters (bilirubin, creatinine levels and INR) collected at admission and determined by using the UNOS Internet site MELD calculator (http://www.unos.org/).

### 2.3. Statistical Analysis

General linear model was used to calculate mean age-adjusted values of MELD score, bilirubin, creatinine, and INR levels by SBP status. Age-adjusted binary logistic regression was used to assess the association of SBP with sex, etiology, and case-fatality rate. Multivariable-adjusted logistic regression was used to assess the independent association of SBP with MELD score. Odds ratios (OR), 95% confidence intervals (CI), and *P* values (*P*) were calculated. The Statistical Package for Social Sciences (SPSS, version 15.0, Chicago, Il, USA) was used for all the statistical analysis.

## 3. Results

Our study included 256 patients (199 men and 57 women) diagnosed with liver cirrhosis and ascites, of whom 64 (25%) were complicated cases with SBP. The etiologic factors of cirrhosis included alcoholic liver disease (*n* = 136, 53.1%), viral hepatitis B (*n* = 76, 29.7%), viral hepatitis C virus (*n* = 6, 2.3%), viral hepatitis with alcohol use (*n* = 12, 4.7%), and other etiologies (*n* = 26, 10.2%). Of 64 cases with SBP, the etiology of cirrhosis was alcohol consumption in 36 cases (56.3%), HBV infection in 15 cases (23.4%), HCV infection in 5 cases (7.8%), alcohol + HBV in 1 case (1.6%), and other etiologies in 7 cases (10.9%).

The characteristics of the two groups of patients, with SBP and without SBP, are summarized in Table. There were no significant differences with regard to age (54.6 versus 54.2 years, *P* = 0.84), sex (men, 81.2% versus 82.8%, *P* = 0.88), and diabetes mellitus (27.6% versus 31.3, *P* = 0.56) between two groups. Alcohol-related etiology was the most common causes of cirrhosis in both groups (52.1% versus 56.3%, *P* = 0.11). MELD score was significantly higher in SBP patients than non-SBP patients (age-adjusted mean score: 23.2 versus 19.5, *P* = 0.003). Conversely, there were no significant differences in mean levels of bilirubin, creatinine, and INR (*P* = 0.37, *P* = 0.22 and *P* = 0.36, resp.). The prevalence of SBP was significantly higher among patients with a higher MELD score (≥25): 37.5% versus 24.5%, *P* = 0.013.

In multivariable-adjusted logistic regression models controlling for age, sex, diabetes, and etiology, there was evidence of a statistically significant difference in SBP rates by MELD score: the OR for developing SBP by each MELD point was 1.06 (95% CI = 1.02–1.09, *P* = 0.002) (not shown in Table 1).

Multivariable-adjusted logistic regression models were run with introduction of bilirubin, creatinine, and INR levels; there was no evidence of statistically significant findings (for bilirubin: OR = 1.01, 95% CI = 0.97–1.06, *P* = 0.483; for creatinine: OR = 1.28, 95% CI = 0.71–2.29, *P* = 0.411; for INR: OR = 0.92, 95% CI = 0.77–1.09, *P* = 0.326) (not shown in Table 1).

The main causes of death among SBP patients were variceal bleeding and hepatorenal syndrome (27% each), liver failure (20%), hepatopulmonary syndrome (13%), and other causes of death (13%). Conversely, the main causes of death among non-SBP patients were variceal bleeding (30%), liver failure (22%), hepatorenal syndrome (10%), hepatopulmonary syndrome (5%), and other causes of death (33%).

Case-fatality rate was significantly higher in SBP patients than non-SBP patients (23.4% versus 12.0%, *P* = 0.024). MELD score was significantly higher in fatal cases versus nonfatal patients (mean age-adjusted score was 32.7 versus 18.4 overall, 34.8 versus 18.0 in SBP patients, and 32.0 versus 18.5 in non-SBP patients; all *P* < 0.001). Furthermore, the mean MELD score was significantly different in patients who died in the group of SBP compared to those who died in the group without SBP (*P* = 0.01).

## 4. Discussion

Our transversal study evaluated the relationship between the MELD score and the development of SBP in the consecutive patients with cirrhosis and ascitis. We found that a higher MELD score was independently associated with a higher risk of SBP. Also, we concluded that MELD score was a significant predictor of hospital mortality in patients with SBP.

The prevalence of SBP as a complication of the ascitic cirrhotic patients included in our study was 25%. It was not much different from the other studies conducted in USA and the European countries [9, 10, 21]. This may be due to relatively high percentage of alcoholic cirrhosis in our country, which is more disposed to develop SBP. Lata et al. reported that the prevalence of SBP is higher in the cirrhosis patients whose ascites is caused by alcohol consumption rather than by viral hepatitis [9], whereas Kaymakoglu et al. came to the opposite conclusion [21]. In the present study, SBP is more frequent in alcoholic-related cirrhosis patients.

A main finding was the strong and statistically significant association of MELD score with SBP, indicating that MELD score may be a useful tool to predict the development of SBP. The patients with moderate to high MELD scores have a substantially greater risk of SBP. The other studies reported the same results; in the study of Malinchoc et al. the mean MELD score of 19.1 in patients with ascites without SPB is lower than the mean MELD score (24.8) of patients with SBP [22], while Gayatri et al. conducted that severe liver cirrhosis with MELD score ≥18 was associated with an increase risk of SBP [23]. Liver cirrhosis with high MELD scores is associated with increased intestinal permeability, which increases the risk for bacterial translocation. Moreover, immunological clearance is decreased in advanced cirrhosis [24]. Therefore, SBP occurs more frequently in patients with advanced cirrhosis.

The MELD score has a predictive value in hospital mortality in patients with end-stage liver disease of diverse etiologies and severity [17, 18]. This prognostic ability of MELD is also confirmed in our study. However, there are few published data, about predictive ability of MELD in hospital mortality in SBP [25]. Our finding that MELD score is an independent predictor of hospital mortality in SBP is in the same line with results of the study of Nobre et al. [25].

The fact that we found also higher level of MELD score in patients with fatal outcome reflects its predictive value based upon biochemistry findings, which combined in this way clearly show the gravity of cirrhotic liver disease. If we take into consideration the values of INR, creatinine and bilirubin separately, we do not find the same statistical predictive significance.

Presence of SBP may be also considered an important sign of deterioration of the liver disease with more frequent fatal outcome, as seen in our cohort of patients, due to their disposal to develop hepatorenal syndrome. The renal failure, due to further reduction of effective circulating volume, is still a common cause of death in the patients with SBP [26].

The serum creatinine has been shown to have a determinant impact on the prognosis of cirrhosis [27]. This parameter is not included in the Child-Turcotte-Pugh score. On the other hand, the MELD score has other advantages when compared with the Child-Pugh score. First, it uses objective parameters second, its objective parameters are less subject to center-to-center variability such as the Child classification, and, third, the MELD score increases as the three constituent parameters deteriorate, whereas the individual scoring elements in the Child score remain fixed once a defined threshold has been reached [28].

Our study may have several limitations. We assessed potential confounders such as age, sex, diabetes, and etiologic factors of cirrhosis, none of which substantially altered the relationship between MELD score and SBP. However, it remains possible that some unrecognized potential confounders may in fact exist and could have influenced the results. Furthermore, the diagnostic paracentesis in all patients was performed upon hospital admission and no information was available with regard to the time elapsed since the onset of SBP. Also, we excluded from the study the patients who were receiving PPI because the acid-suppressive therapy may lead to small intestinal bacterial overgrowth and subsequent bacterial translocation into the peritoneal cavity and finally increase the risk of SBP [29].

The clinical spectrum of SBP is quite variable, ranging from sings of peritonitis to complete absence of symptoms; therefore the diagnosis relies on the high index of suspicions and the examination of the ascitic fluid [30]. Our study supports the fact that MELD score has a high predictive value for the SBP development and fatal outcome in liver cirrhosis. These findings can be useful for increasing suspicions for SBP development in hospitalized cirrhotic patients with elevated MELD score.

## Figures and Tables

**Table 1:** Association of selected risk factors with spontaneous bacterial peritonitis (SBP) in hepatic cirrhosis patients with ascites.

Variable	Without SBP (*N* = 192)	With SBP (*N* = 64)	*P* value
Age (years)	54.56 (52.73–56.38)*	54.20 (51.04–57.36)*	0.849
MELD score	19.49 (18.38–20.60)	23.20 (21.27–25.12)	0.001
MELD group			0.046^‡^
≤15	64 (33.3)^†^	12 (18.8)^†^	—
16–24	81 (42.2)	28 (43.8)	0.111
≥25	47 (24.5)	24 (37.5)	0.013
Bilirubin (mg/dL)	5.41 (4.38–6.44)	6.34 (4.56–8.13)	0.373
Creatinine (mg/dL)	0.99 (0.92–1.06)	1.08 (0.96–1.20)	0.229
INR (units)	2.57 (2.24–2.90)	2.27 (1.70–2.84)	0.364
Sex			
Women	41 (21.4)	16 (25.0)	0.887^‡^
Men	151 (78.6)	48 (75.0)	
Etiology			
Alcohol	100 (52.1)	36 (56.3)	0.111^‡^
Other^§^	92 (47.9)	28 (43.7)	
Diabetes			
No	139 (72.4)	44 (68.7)	0.560^‡^
Yes	53 (27.6)	20 (31.3)	
Death			
No	169 (88.0)	49 (76.6)	0.024^‡^
Yes	23 (12.0)	15 (23.4)	

*Mean age-adjusted values and 95% confidence intervals (in parenthesis) from the general linear model.

^†^Number and column percentages (in parenthesis).

^‡^Age-adjusted *P* values from binary logistic regression.

^§^HBV, HCV, or alcohol + virus.
